# Xác định đột biến gen liên quan đến tính kháng thuốc moxifloxacin của chủng vi khuẩn *mycobacterium Tuberculosis* tại hà nội năm 2020 – 2022

**DOI:** 10.51403/0868-2836/2023/1461

**Published:** 2024-03-20

**Authors:** Khiếu ThỊ Thúy Ngọc, Nguyễn Văn Hưng, Đinh ThỊ Hương, Lê Thị Nam, Vũ Ngọc Trung, Đoàn Thu Hà, Nguyễn Văn Khiêm, Nguyễn Kim Cương, Nguyễn Bình Hòa, Đinh Văn Lượng, Nguyễn Thụy Thương Thương, Timothy M Walker, Guy Thwaites

**Affiliations:** 1 Bệnh viện Phổi Trung ương (NLH), Hà NỘi; 2 Trường Đại học Y Dược, Đại học Quốc gia Hà NỘi (VNU-UMP); 3 Bệnh viện Phổi Hà NỘi (HLH); 4 Đơn vỊ Nghiên cứu Lâm sàng Đại học Oxford (OUCRU), Thành phố Hồ Chí Minh

**Keywords:** *Mycobacterium tuberculosis*, giải trình tự toàn bộ hệ gen, lao đa kháng thuốc (MDR-TB), *Mycobacterium tuberculosis*, Fluoroquinolones, mutations, Moxifloxacin (MFX), sequencing, multi-drug resistant tuberculosis (MDR-TB)

## Abstract

Nguy cơ kháng với các thuốc điều trỊ lao là vấn đề toàn cầu gây ảnh hưởng đến an toàn sức khỏe và mục tiêu kết thúc bệnh lao vào năm 2035. Tính kháng thuốc của vi khuẩn lao liên quan đến sự phát sinh các đột biến một cách ngẫu nhiên ở các gen nằm trong vùng nhân của tế bào vi khuẩn. Nghiên cứu này áp dụng phương pháp giải trình tự toàn bộ hệ gen vi khuẩn lao (WGS) nhằm phân tích các đột biến liên quan đến tính kháng thuốc lao Moxifloxacin (MFX). Kết quả nghiên cứu đã phân lập được 82 chủng *M. tuberculosis* có đầy đủ kết quả kháng sinh đồ kiểu hình và giải trình tự toàn bộ hệ gen, trong đó có 16 chủng kháng MFX. Kết quả giải trình tự gen *gyrA* cho thấy, 37,5% (6/16) chủng kháng MFX mang ít nhất 1 đột biến kháng MFX và chỉ có 1,5% (1/66) chủng nhạy MFX mang đột biến kháng. Đột biến thường gặp nhất được phát hiện là trên gen gyrA_p.Asp94Gly (67%), đột biến chỉ xảy ra ở dòng lineage 2 - Beijing (Bắc Kinh) liên quan đến kháng thuốc. Chẩn đoán phân tử dựa trên đột biến gen *gyr*A có độ nhạy 37,5% và độ đặc hiệu 98,5%. Đây là cơ sở quan trọng để xem xét việc ứng dụng giải trình tự gen phát hiện nhanh *M. tuberculosis* kháng thuốc, tiến đến cá thể hóa điều trỊ bệnh lao.

## Abstract (en)

The risk of resistance to tuberculosis drugs is a global problem that affects health safety and the goal of ending tuberculosis by 2035. Drug resistance of tuberculosis bacteria involves the emergence of unique mutations. randomly in genes located in the nucleus of bacterial cells. This study applies the MTB whole genome sequencing (WGS) method to analyze mutations related to TB drug resistance to Moxifloxacin (MFX). The results of the study were to isolate 82 strains of *M. tuberculosis* with complete phenotypic antibiogram results and whole genome sequencing, of which 16 strains were resistant to MFX. The gyrA gene sequencing results showed that 37.5% (6/16) of MFX-resistant strains carried at least 1 MFX resistance mutation and only 1.5% (1/66) of MFX-sensitive strains carried a resistance mutation. The most common mutations detected was in the gyrA_p.Asp94Gly gene (67%), the mutation only occurred in lineage 2 - Beijing related to drug resistance. Molecular diagnosis based on gyrA gene mutations has a sensitivity of 37.5% and a specificity of 98.5%. This is an important basis for considering the application of gene sequencing to quickly detect drug-resistant *M. tuberculosis*, moving towards individualizing tuberculosis treatment nationwide.

## I Ðặt Vấn Ðề

Bệnh lao (TB) là một bệnh truyền nhiễm gây bởi *Mycobacterium tuberculosis* và hiện đang là một trong những nguyên nhân chính gây tử vong do các bệnh truyền nhiễm trên khắp thế giới. Tác nhân gây bệnh lao, *Mycobacterium tuberculosis*, chính thức được xác định và mô tả vào năm 1882. Ðây là công trình nghiên cứu của Robert Koch (1843 - 1910). Ðể ghi nhận công lao của ông, người ta còn gọi trực khuẩn lao gây bệnh trên người là trực khuẩn Koch (Robert Koch) [1]. Trong thế kỷ 20, bệnh lao đã giết chết khoảng 100 triệu người. Từ năm 1943, streptomycin, thuốc chống lao đầu tiên được phát hiện và đưa vào sử dụng điều trỊ có hiệu quả. Sau đó, hàng loạt thuốc chống lao khác ra đời đã mở ra một kỷ nguyên mới trong điều trỊ bệnh lao, cũng như đã cứu chữa cho hàng triệu bệnh nhân lao khỏi căn bệnh nguy hiểm này. Cùng với sự phát triển của đại dỊch HIV/AIDS, dỊch tễ lao toàn cầu đã gây ra những thách thức rất lớn cho cuộc chiến chống lao của loài người, đó là sự tương tác giữa lao và đại dỊch HIV/AIDS và sự phát triển của các chủng vi khuẩn lao kháng thuốc [2].

Việt Nam thuộc nhóm 30 quốc gia có gánh nặng bệnh lao và lao kháng thuốc cao nhất thế giới. Năm 2022, ước tính có 172.000 người mắc lao với khoảng 13.000 ca tử vong [3, 4]. Số mắc lao kháng rifampicin hoặc đa kháng thuốc khoảng 8.200 trường hợp. Hướng tới mục tiêu thanh toán bệnh lao vào năm 2035, Việt Nam đang tiến đến áp dụng công nghệ giải trình tự toàn bộ hệ gen phát hiện đột biến kháng thuốc để chẩn đoán lao kháng thuốc, từ đó tối ưu hóa phác đồ điều trỊ cá thể với từng bệnh nhân lao. Các thuốc lao hàng một bao gồm Isoniazid (INH), Rifampicin (RIF), Pyrazinamid (PZA), Ethambutol (EMB). Ðây là các thuốc được sử dụng trong phác đồ điều trỊ lao nhạy cảm phổ biến hiện nay. Các thuốc lao hai là các thuốc nhóm fluoroquinolone (Ciprofloxacin, Levofloxacin, Moxifloxacin), aminoglycoside và polypeptide (Amikacin, Kanamycin, Capreomycin), thioamide (Ethionamide, Prothionamide), cycloserine và para-aminosalicylic acid [5]. Theo quyết đỊnh số 1314/QÐ-BYT ngày 24 tháng 3 năm 2020 của Bộ Y tế, bệnh lao kháng thuốc được phân loại theo tiền sử điều trỊ, bao gồm: Kháng đơn thuốc là bệnh lao chỉ kháng với duy nhất một thuốc chống lao hàng một khác Rifampicin; kháng nhiều thuốc bệnh lao kháng với từ hai thuốc chống lao hàng một trở lên mà không kháng với Rifampicin; lao kháng Rifampicin là bệnh lao kháng với Rifampicin, có hoặc không kháng thêm với các thuốc lao khác kèm theo (có thể là kháng đơn thuốc, kháng nhiều thuốc, đa kháng thuốc hoặc siêu kháng thuốc); đa kháng thuốc (MDR-TB) là bệnh lao kháng đồng thời với ít nhất hai thuốc chống lao là Isoniazid và Rifampicin; tiền siêu kháng là bệnh lao đa kháng có kháng thêm với hoặc bất cứ thuốc nào thuộc nhóm Fluoroquinolone hoặc với ít nhất một trong ba thuốc tiêm hàng hai (Capreomycin, Kanamycin, Amikacin, chứ không đồng thời cả 2 loại thêm); siêu kháng thuốc (XDR-TB) là bệnh lao đa kháng thuốc có kháng thêm với bất cứ thuốc nào thuộc nhóm Fluoroquinolone và với ít nhất một trong ba thuốc tiêm hàng hai (Capreomycin, Kanamycin, Amikacin).

Việc xác đỊnh tính kháng thuốc ở quần thể vi khuẩn gây bệnh lao, đặc biệt là tính kháng INH, RIF và MFX sẽ giúp bác sĩ lâm sàng chẩn đoán đúng và lựa chọn phác đồ điều trỊ bệnh lao một cách chính xác và hiệu quả.

Sự lưu hành của chủng *M. tuberculosis* ở mỗi nước có đặc thù riêng, tỷ lệ kháng với các thuốc lao ở mỗi khu vực rất khác nhau. Ða dạng đột biến kháng các thuốc lao trong quần thể vi khuẩn *M. tuberculosis* phân lập từ bệnh nhân lao tại Việt Nam cũng có thể có sự khác biệt [2, 4, 6, 7]. Do đó, mục tiêu của nghiên cứu này là xác đỊnh đột biến kháng MFX của chủng *M. tuberculosis* tại Việt Nam và mối liên quan giữa đa dạng đột biến kháng thuốc này với các dòng *M. tuberculosis* đang lưu hành. Kết quả nghiên cứu sẽ cung cấp dữ liệu đột biến kháng thuốc Fluoroquinolones của *M. tuberculosis* tại Việt Nam, là tiền đề quan trọng trong việc áp dụng công nghệ chẩn đoán phân tử hiện đại như giải trình tự gen đích để phát hiện nhanh vi khuẩn *M. tuberculosis* kháng thuốc.

## II Phương Phàp Nghién Cứu

### 2.1 Ðối tượng nghiên cứu

Mẫu vi khuẩn *M. tuberculosis* được phân lập từ bệnh nhân lao có kết quả xét nghiệm Xpert MTB dương tính kháng RIF đến khám tại Bệnh viện Phổi Trung ương và Bệnh viện Phổi Hà Nội trong giai đoạn từ năm 2020 – 2022.

#### Tiêu chuẩn lựa chọn mẫu

Mẫu đờm được thu thập từ bệnh nhân/người nghi lao phổi mới từ 18 tuổi trở lên chưa điều trỊ với thuốc lao hoặc điều trỊ lao dưới hoặc bằng 30 ngày.

Mẫu đờm đạt chất lượng và có thông tin đầy đủ đi kèm bao gồm: Họ và tên bệnh nhân, tuổi, giới, khoa, tình trạng điều trỊ với thuốc lao tại thời điểm thu mẫu, tiền sử bệnh, loại bệnh phẩm.

#### Tiêu chuẩn loại trừ

Mẫu đờm thu thập từ người nghi lao có tiền sử điều trỊ lao trên 30 ngày hoặc người nghi lao dưới 18 tuổi.

Mẫu đờm không đạt chất lượng, không có thông tin đầy đủ thông tin bao gồm: Họ và tên bệnh nhân, tuổi, giới, khoa, tình trạng điều trỊ với thuốc lao tại thời điểm thu mẫu, tiền sử bệnh, loại bệnh phẩm.

Mẫu đờm từ người nghi lao không đồng ý tham gia nghiên cứu.

### 2.2 Ðịa điểm và thời gian nghiên cứu

Nghiên cứu được thực hiện tại Bệnh viện Phổi Trung ương và Bệnh viện Phổi Hà Nội từ tháng 11/2020 đến hết tháng 12/2022.

Ðịa điểm thực hiện xét nghiệm: Khoa Vi sinh và Labo lao chuẩn Quốc gia, Bệnh viện Phổi Trung ương.

### 2.3 Thiết kế nghiên cứu

Nghiên cứu mô tả cắt ngang.

### 2.4 Cỡ mẫu nghiên cứu

Tổng số 100 mẫu đờm của bệnh nhân lao có kết quả xét nghiệm Xpert MTB dương tính kháng RIF đến khám tại Bệnh viện Phổi Trung ương và Bệnh viện Phổi Hà Nội trong giai đoạn từ năm 2020 – 2022.

### 2.5 Phương pháp chọn mẫu

Thu thập toàn bộ các mẫu đờm của bệnh nhân lao thỏa mãn tiêu chí lựa chọn và đồng ý tham gia nghiên cứu.

### 2.6 Biến số nghiên cứu

Nhóm biến số về đặc điểm nhân khẩu họ tên, tuổi, giới tính, tiền sử của bệnh nhân; kết quả xét nghiệm của các chủng vi khuẩn *M. tuberculosis* phân lập được từ mẫu đờm của bệnh nhân theo phân loại nhạy, kháng thuốc, loại gen và vỊ trí đột biến kháng thuốc.

### 2.7 Phương pháp thu thập thông tin

Ðặc điểm nhân khẩu học được thu thập qua phiếu thu thập thông tin của người bệnh ngay từ khi đến khám và thu mẫu xét nghiệm.

Các thông tin về kết quả xét nghiệm được ghi nhận vào sổ xét nghiệm hàng ngày và cập nhật cho nhóm nghiên cứu.

#### Xét nghiệm thực hiện trong nghiên cứu

Mẫu đờm của các bệnh nhân có kết quả xét nghiệm Xpert MTB dương tính kháng RIF được thu thập với số lượng từ 4 - 6ml đờm đặc, nhày mủ. Các mẫu được xử lí mẫu theo quy trình nuôi cấy vi khuẩn lao trên môi trường lỏng MGIT. Nếu kết quả MGIT dương tính sẽ được tiếp tục làm đỊnh danh, kháng sinh đồ để xác đỊnh tính kháng thuốc hàng 1, 2 và các thuốc khác của vi khuẩn lao phân lập được. Các chủng *M.tuberculosis* cũng tiếp tục được tách ADN và thực hiện giải trình tự gen để xác đỊnh tính kháng thuốc và so sánh với kết quả kháng sinh đồ từ chủng MGIT dương tính. Các kỹ thuật xét nghiệm thực hiện theo tài liệu hướng dẫn quy trình thực hành chuẩn các xét nghiệm Vi sinh lao (2018), Bộ Y tế - CTCLQG.

Các chủng *M.tuberculosis* sau khi cấy MGIT dương tính được bảo quản ở -20°C để tiếp tục tách DNA và giải trình tự hệ gene xác đỊnh tính kháng thuốc. *Mycobacterium tuberculosis* (MTB) trong mẫu chủng cấy MGIT dương tính sẽ bỊ bất hoạt bởi sự kết hợp giữa nhiệt và lysozyme. Thành tế bào vi khuẩn sau đó sẽ bỊ phá vỡ bằng phương pháp cơ học. DNA sẽ được làm tinh sạch bằng hạt AMPure XP. Hệ thống tinh sạch Agenecourt AMPure XP PCR* sử dụng công nghệ hạt thuận từ đảo ngược pha rắn (SPRI) của Backman Coulter để tinh sạch DNA chất lượng cao. Agenecourt AMPure XP sử dụng một chất đệm được tối ưu hóa để liên kết có chọn lọc ra các đoạn DNA có chiều dài lớn hơn 100 bp. Sau đó loại bỏ mồi, nucleotide, muối, và các enzyme dư thừa bằng một quy trình rửa đơn giản với ethanol. DNA cuối cùng được thu nhận trong nước deion hoặc đệm TE.

Thực hiện giải trình tự bộ gene vi khuẩn lao bằng hệ thống Illumina MiniSeq được thực hiên theo quy trình số WGS 06 - BVPTW bao gồm các bước chính như sau:

Bước 1: Chuẩn bỊ thư viện bằng kit Nextera XT với mục đích tạo ra các trình tự DNA được cắt ở độ dài từ 300 – 700bp và gắn adaptor ở hai đầu của đoạn gene. Các adaptor này giúp DNA bám trên flow cell trong quá trình đọc, đồng thời cũng là đoạn mồi trong phản ứng và chỉ thỊ phân biệt trình tự từ các mẫu khác nhau.

Bước 2: Chạy chương trình giải trình tự trên máy. Các đoạn DNA được đưa vào và gắn trên bề mặt flow cell. Sau đó, các đoạn DNA sẽ được khuếch đại tạo thành các cụm (cluster).

Bước 3: Hệ thống sẽ giải trình tự dựa trên “kỹ thuật giải trình tự tổng hợp” và sử dụng các dNTP có gắn các tín hiệu huỳnh quang tương ứng với 4 loại nucleotide. Trong mỗi chu kỳ giải trình tự, từng nucleotide có gắn tín hiệu huỳnh quang được gắn vào chuỗi axit nucleic theo nguyên tắc bổ sung trên sợi DNA khuôn. Vì nucleotide được gắn vào như là một chất chấm dứt kéo dài chuỗi, nên phản ứng tạm dừng lại. Sau khi gắn từng nucleotide, tín hiệu huỳnh quang sẽ được ghi lại để xác đỊnh các trình tự của loại nucleotide được được gắn vào DNA.

Bước 4: Quá trình giải trình tự kết thúc sẽ xuất các trình tự (read) trong file với đỊnh dạng fastq.gz và có thể bắt đầu thực hiện phân tích dữ liệu.

Lưu file fastq về ổ lưu trữ nội bộ, ghi nhận ngày chạy, các thông số để đánh giá kết quả chất lượng chạy giải trình tự (thông số hiển thỊ trên máy MiniSeq khi kết thúc quá trình giải trình tự như cluster density, estimate output, passing filter).

Kết quả giải trình tự cho biết đỊnh danh tên loài vi khuẩn, dự báo tính kháng thuốc và xác đỊnh dòng vi khuẩn dựa trên các trình tự gene bằng cách sử dụng phần mềm sinh tin Oxford SP3 predict, Gnomonicus và MyKrobe.

### 2.8 Xử lý và phân tích số liệu

Nhập số liệu vào file excel, sử dụng phần mềm tin sinh Mykrobe, Clockwork và thống kê SPSS để phân tích dữ liệu nghiên cứu.

### 2.9 Ðạo đức nghiên cứu

Nghiên cứu đã được Hội đồng đạo đức trong Nghiên cứu Y sinh học của Bệnh viện Phổi Trung ương cho phép thực hiện theo quyết đỊnh số 21/CT-HDDD ngày 25/5/2020.

## III Kết Quả

### 3.1 Thông tin chung về chủng và đối tượng nghiên cứu

Tổng số 100 bệnh nhân lao có kết quả xét nghiệm Xpert MTB (+) / RIF (+) đến khám tại Bệnh viện Phổi Trung ương và Bệnh viện Phổi Hà Nội trong giai đoạn từ năm 2020 – 2022. Các mẫu đờm được nuôi cấy phân lập và thực hiện kháng sinh đồ thuốc lao hàng 1, 2 từ các mẫu cấy dương tính với MTB, đồng thời thực hiện giải trình tự toàn bộ hệ gen từ chủng cấy dương tính. Kết quả có 82 chủng vi khuẩn *M. tuberculosis* có đủ thông tin kháng thuốc kiểu hình (KSÐ) và kháng thuốc kiểu gen bằng kỹ thuật giải trình tự toàn bộ hệ gen (WGS) được đưa vào phân tích.

Kết quả bảng 1 cho thấy 52% (52 mẫu) được thu thập từ bệnh viện (BV) Phổi Trung ương và 480% (48 mẫu) thu thập từ BV Phổi Hà Nội. Có 78,0% là nam giới và 22,0% là nữ giới đang quản lý điều trỊ tại hai BV ở Hà Nội. Mẫu có kết quả soi đờm dương tính từ 1+ trở lên chiếm tỷ lệ 77,0% và 23,0% là dương tính với số lượng AFB ít từ 1 - 9 AFB /100 vi trường. Các mẫu có kết quả Xpert MTB (+)/ RIF (+) chiếm 98,0 % và 2 mẫu có kết quả Xpert MTB (+)/ RIF không xác đỊnh tính kháng thuốc. Các mẫu được thực hiện nuôi cấy trên môi trường lỏng MGIT 960 trong đó có 95 mẫu cấy dương tính MTB, 3 mấu cấy dương tính NTM và 2 mẫu cấy chủng nhiễm. Các mẫu cấy dương tính MTB được làm xét nghiệm kháng sinh đồ với thuốc lao hàng 1, 2 và tách DNA để làm giải trình tự gen. Trong đó có 82 mẫu có đầy đủ kết quả kháng thuốc kiểu hình và kháng thuốc kiểu gen để đưa vào phân tích.

Kết quả kháng thuốc kiểu hình và kháng thuốc kiểu gen của 82 mẫu nghiên cứu theo dòng vi khuẩn được thể hiện ở bảng 2. Các kiểu kháng/nhạy thuốc ở trên được xác đỊnh bằng kháng sinh đồ dựa trên trên nuôi cấy trên môi trường lỏng (Mycobacteria_growth_indicator MGIT) tại khoa Vi sinh và Labo Lao chuẩn Quốc gia, Bệnh viện Phổi Trung ương. Kết quả cho thấy tỷ lệ chủng kháng đơn hoặc nhiều thuốc chiếm 14,6% (12/82), tỷ lệ chủng kháng đa thuốc (MDR-TB) và kháng RIF (RR-TB) là 69,5% (57/82) và tỷ lệ chủng tiền/siêu kháng thuốc (PreXDR/ XDR – TB) là 15,9% (13/82). Kết quả giải trình tự toàn bộ hệ gen chủng M. tuberculosis trong nghiên cứu này cho thấy quần thể mẫu nghiên cứu thuộc 3 dòng với tỷ lệ tương ứng là: dòng 1- EAI (4,9%), dòng 2 – Beijing (86,6%) và dòng 4 -T, H, LAM (8,5%).

### 3.2 Ðột biến kháng MFX ở vi khuẩn M. tuberculosis

Kết quả có 16 mẫu kháng thuốc MFX kiểu hình, trong đó có 10 mẫu không có đột biến trên gene gyrA và 6 mẫu có các đột biến trên gene gyrA, (4/6) chủng kháng MFX mang ít nhất 1 đột biến kháng MFX và chỉ có 1,5% (1/66) chủng nhạy mang đột biến kháng. Ðột biến thường gặp nhất được phát hiện là trên gen gyrA_p.Asp94Gly (67%), đột biến chỉ xảy ra ở dòng lineage 2 - Beijing (Bắc Kinh) liên quan đến kháng thuốc. Ðột biến ở các codon khác trên gen gyrA được phát hiện ở 1 chủng tại 4 vỊ trí khác nhau trên gen gyrA là gyrA_p.Ala90Val, gyrA_p.Glu21Gln, gyrA_p. Gly668Asp, gyrA_p.Ser95Thr, trong đó có 1 vỊ trí liên quan đến kháng thuốc theo công bố của WHO là gyrA_p.Ala90Val [8].

### 3.3 Mối liên quan giữa các dòng M. tuberculosis với tần suất và đa dạng đột biến

Tỷ lệ chủng kháng MFX là 16/82 (19,5%), chủng nhạy MFX là 66/82 (80,5%). Kháng thuốc MFX kiểu gen xảy ra ở 6/16 chủng MTB có kháng thuốc kiểu hình và đột biến kháng MFX kiểu gen chỉ xảy ra ở chủng thuộc dòng lineage 2 và không có kháng thuốc kiểu hình. Ðô nhạy và độ đặc hiệu chung cho giải trình tự gen dự báo kháng thuốc MFX là 37,5% và 98,5%.

## IV Bàn Luận

Trong nghiên cứu của chúng tôi, số lượng bệnh nhân là nam chiếm đa số (78,0%), gần gấp bốn nữ (22,0%) (Bảng 1). Tỷ lệ này phù hợp với hầu hết nhiều nghiên cứu trong nước và trên thế giới đã ghi nhận: Số lượng nam mắc bệnh lao luôn cao hơn so với nữ [9]. Tác giả Nguyễn Huy Ðiện (2010), nghiên cứu tính kháng thuốc của MTB phân lập được ở 218 bệnh nhân tràn dỊch màng phổi (TDMP) do lao. Trong số đó, nam chiếm ưu thế với tỷ lệ 81,7%. Nghiên cứu trên những bệnh nhân lao phổi tái phát tại Bệnh viện Phổi Trung ương và bệnh viện 74 Trung ương, Nguyễn Thu Hà (năm 2011 - 2012) đã ghi nhận sự phân bố tỷ lệ bệnh nhân nam lao phổi tái phát cao gấp từ 3 – 5 lần so với bệnh nhân nữ. Số liệu từ bảng 2 về chủng *M. tuberculosis* nghiên cứu theo kiểu kháng thuốc và theo dòng vi khuẩn cho thấy dòng 2 chiếm 87,0% và tiền siêu kháng thuốc là 57%. Tỷ lệ chủng kháng đơn hoặc nhiều thuốc (không bao gồm INH và RIF) chiếm 14,6% (12/82), tỷ lệ chủng kháng đa thuốc (MDR- TB) và kháng RIF (RR-TB) là 69,5% (57/82) và tỷ lệ chủng tiền/siêu kháng thuốc (PreXDR/ XDR – TB) là 15,9% (13/82), trong đó có 1 chủng siêu kháng thuốc (XDR-TB), chủng này kháng đồng thời INH, RIF, SM ZA, MFX và KM (theo phân loại kháng thuốc WHO năm 2020).

Dựa trên kết quả phân loại thực hiện bằng kỹ thuật giải trình tự toàn bộ hệ gen chủng *M. Tuberculosis*, 82 chủng trong nghiên cứu này được xác đỊnh thuộc 3 dòng chính là dòng 1- EAI (4,9%), dòng 2 – Beijing (86,6%) và dòng 4 -T, H, LAM (8,5%). Trong đó dòng 2 – Beijing chiếm đa số và tỷ lệ đa kháng thuốc MDR-TB và RR-TB xảy ra nhiều hơn (82,9% và 85,4%) ở các chủng dòng 2 – Beijing.

Nghiên cứu này tuân thủ nguyên tắc của WHO rằng bất kỳ đột biến gyrA nào đáp ứng các tiêu chí về tính kháng MFX theo phân loại 1 là có liên quan đến kháng thuốc hoặc ở mức độ 2 là liên quan ở mức độ vừa cũng được xem là kháng với MFX. Do quy tắc này nhất quán với dữ liệu MIC đã công bố và được xem là các chỉ thỊ tạm thời cho tính kháng MFX [3, 7]. Quy tắc này cũng được áp dụng để nâng cấp gyrB E501D lên đột biến nhóm 2 về tính kháng MFX, vì nó đáp ứng các tiêu chí cho nhóm 1 trong các chủng phân lập kháng MFX [3, 10]. Tuy nhiên, nghiên cứu này không phát hiện có đột biến trên gen gyrB.

Ðộ nhạy và độ đặc hiệu để dự đoán các kiểu hình kháng MFX được tính toán trong nghiên cứu này có lẽ không đại diện cho hiệu quả thực tế của 16 đột biến kháng FQs trong môi trường phòng thí nghiệm lâm sàng. Ðầu tiên, chỉ những đột biến có tần số alen ≥ 90% trong các mẫu quần thể hỗn hợp mới được đưa vào tập dữ liệu được phân tích, điều này có thể dẫn đến đánh giá thấp độ nhạy, vì tính dị kháng đóng vai trò quan trọng trong khả năng kháng FQ. Thứ hai, tài liệu cập nhật của WHO năm 2021 [8], độ nhạy và độ đặc hiệu của xét nghiệm giải trình tự gen dự báo kháng thuốc MFX (CB 1,0µg/ml) là 84,8% và 94%. Ðồng thời, nồng độ thuốc MFX khi làm kháng sinh đồ MTB trên môi trường lỏng MGIT được khuyến cáo ở hai mức độ nồng độ tới hạn (Critical concentrations - CC) là 0,25µg/ml và điểm dừng lâm sàng (clinical breakpoints - CB) là 1,0µg/ml. Nghiên cứu này sử dụng nồng độ MFX CC (0,25µg/ml), dẫn đến sự phù hợp kém giữa các đột biến và tính kháng kiểu hình đối với các chủng nghiên cứu. Ðiều này lý giải cho việc độ nhạy của giải trình tự gen trong chẩn đoán kháng MFX cho 82 mẫu khá thấp. Ðộ nhạy là 37,5% và độ đặc hiệu là 98,5%.

Trên thế giới, tần suất và tỷ lệ đột biến gen gyrA nói chung và các đột biến chính như gyrA_p.Gly668Asp, gyrA_p.Glu21Gln và gyrA_p.Ser95Thr khác nhau theo khu vực đỊa lý và quốc gia [11]. Trong nghiên cứu này do số lượng chủng kháng MFX thuộc dòng 2 không nhiều (6/16 chủng), sự khác nhau về tần suất xuất hiện đột biến kháng thuốc và đa dạng đột biến giữa các dòng vi khuẩn *M. tuberculosis* không có ý nghĩa thống kê. Tuy nhiên số liệu cho thấy, đột biến kháng MFX phát hiện ở chủng dòng 2 – Beijing có mức độ đa dạng cao, số codon bỊ ảnh hưởng lớn nhất và bao gồm cả đột biến nằm trong và ngoài vùng nóng, tần suất đột biến tại 3 codon gyrA_p. Gly668Asp, gyrA_p.Glu21Gln và gyrA_p. Ser95Thr cao nhất. Như vậy đa dạng đột biến gen gyrA có mối liên quan với dòng vi khuẩn *M. tuberculosis*, trong đó dòng lineage 2 - Beijing là dòng phổ biến ở khu vực châu Á [3, 12]. Việc thực hiện giải trình tự hệ gen vi khuẩn *M.tuberculosis* từ chủng vi khuẩn sau khi nuôi cấy dương tính so sánh với kết quả kháng sinh đồ truyền thống cho phép rút ngắn thời gian chẩn đoán và dự báo phác đồ điều trỊ phù hợp với từng bệnh nhân.

## V Kết Luận

Tỷ lệ kháng MFX kiểu hình trong quần thể nghiên cứu là 19,5% trong đó 6/16 chủng (37,5%) mang gen đột biến liên quan đến kháng thuốc được phát hiện bằng xét nghiệm giải trình tự toàn bộ hệ gen. Ðột biến thường gặp nhất được phát hiện là trên gen gyrA_p. Asp94Gly (67%), đột biến chỉ xảy ra ở dòng lineage 2 - Beijing (Bắc Kinh) liên quan đến kháng thuốc. Ðộ nhạy và độ đặc hiệu trong phát hiện kháng MFX bằng xét nghiệm giải trình tự hệ gen *M. tuberculosis* là 37,5% và 98,5%.

Kết quả nghiên cứu này góp phần cung cấp dữ liệu đột biến các gen liên quan đến kháng MFX theo dòng vi khuẩn *M. tuberculosis* lưu hành ở Hà Nội, Việt Nam. Tỷ lệ chủng kháng Moxifloxacin (MXF) mang đột biến khá cao cho thấy hiệu quả khi áp dụng công nghệ phân tử hiện đại như giải trình tự gen đích trong việc phát hiện nhanh và chính xác tính kháng MFX ở vi khuẩn *M. tuberculosis*, chẩn đoán sớm các chủng vi khuẩn tiền siêu kháng thuốc (XDR-TB), tiến tới cá thể hóa phác đồ điều trỊ, tăng tỷ lệ điều trỊ thành công bệnh lao tại Việt Nam.

### Lời cảm ơn

Nghiên cứu này được thực hiện với sự hỗ trợ kinh phí của nhiệm vụ hợp tác Quốc tế về Khoa học và Công nghệ theo nghỊ đỊnh thư giữa Bệnh viện Phổi Trung ương, Ðơn vỊ Nghiên cứu Lâm sàng Ðại học Oxford (OUCRU) Tp Hồ Chí Minh, quỹ MRC Newton của Vương quốc Anh mã số MR/R006319/1 và Bộ Khoa học và Công nghệ, đề tài “Giải trình tự gen vi khuẩn lao nhằm dự báo tính nhạy cảm với thuốc kháng lao ở bệnh nhân lao kháng đa thuốc tại Việt Nam”, mã số đề tài NÐT.81.GB/20. Chủ nhiệm đề tài là PGS. TS. BS. Nguyễn Văn Hưng, Bệnh viện Phổi Trung ương và đồng chủ nhiệm là TS. BS. Guy Thwaites, Ðơn vỊ Nghiên cứu Lâm sàng Ðại học Oxford (OUCRU) Tp Hồ Chí Minh.

## Figures and Tables

**Bảng 1:** Đặc điểm mẫu nghiên cứu (tiếp)

Nội dung	Mẫu xét nghiệm (n)	Tỷ lệ (%)
Điểm nghiên cứu		
Bệnh viện Phổi Trung ương	52	52,0
Bệnh viện Phổi Hà Nội	48	48,0
Giới tính		
Nam	78	78,0
Nữ	22	22,0
Tuổi trung bình		
Dưới 30 tuổi	18	18,0
Từ 30 - 39 tuổi	18	18,0
Từ 40 - 49 tuổi	26	26,0
Từ 50 - 59 tuổi	17	17,0
Từ 60 - 69 tuổi	17	17,0
Từ 70 tuổi +	4	4,0
Kết quả soi Đờm		
AFB ít	23	23,0
1+	35	28,0
2+	14	14,0
3+	28	28,0
Kết quả Xpert MTB/RIF		
MTB (+) / RIF (+)	98	98,0
MTB (+) / RIF không xác Định	2	2,0
Kết quả nuôi cấy		
MTB	95	95,0
NTM	3	3,0
Chủng nhiễm	2	2,0
Mẫu cấy MTB có kết quả kháng thuốc kiểu hình và kiểu gen		
Có kết quả kháng thuốc	82	82,0
Không có kết quả kháng thuốc	18	18,0

**Bảng 2:** Chủng *M. tuberculosis* nghiên cứu theo kiểu kháng thuốc và theo dòng vi khuẩn

Kiểu hình kháng thuốc	Dòng *M. tuberculosis*			Tổng	Tỷ lệ (%)
	Dòng 1	Dòng 2	Dòng 4		
Kháng đơn/nhiều thuốc	1	11	0	12	14,6
Kháng đa thuốc	3	47	7	57	69,5
Siêu/tiền siêu kháng	0	11	2	13	15,9
Tổng số	4 (4,9%)	69 (86,6%)	9 (8,5%)	82	100,0

**Bảng 3:** Đột biến gen ở vi khuẩn *M. tuberculosis* kháng và không khàng MFX

Gen kháng thuốc	Vị trí đột biến	Phân loại	Tần suất gặp phải trong chủng
MFX kiểu hình kháng thuốc và kiểu gen kháng thuốc, (n = 6)			
gyrA	gyrA_p.Ala90Val	1) Assoc w R	1
	gyrA_p.Asp94Gly	1) Assoc w R	4
	gyrA_p.Asp94Asn	1) Assoc w R	1
MFX kiểu hình nhạy (S), kiểu gen kháng (R), (n = 1)			
gyrA	gyrA_p.Ala90Val	1) Assoc w R	1

**Bảng 4:** Tần suất đột biến các gen liên quan đến kháng MFX ở các dòng *M. tuberculosis* và giá trỊ chẩn đoán phân tử dựa vào đột biến

	Chủng có/ không mang đột biến kháng MFX					
Dòng *M.tuberculosis*	Chủng nhạy MFX 66/82 = 80,5%		Chủng kháng MFX 16/82 = 19,5%		Giá trị chẩn đoán	
	Có	Không	Có	Không	Độ nhạy (%)	Độ đặc hiệu (%)
Lineage 1	0	4	0	0		
Lineage 2	1	54	6	10	37,5	98,5
Lineage 4	0	7	0	0		
Tổng	1	65	6	10		

## References

[1] Cole ST, Brosch R, Parkhill J (1998). Deciphering the biology of *Mycobacterium tuberculosis* from the complete genome sequence. Nature.

[2] Nguyen VAT, Bañuls AL, Tran THT (2016). *Mycobacterium tuberculosis* lineages and anti-tuberculosis drug resistance in reference hospitals across Viet Nam. BMC microbiology.

[3] Nguyen HQ, Nguyen NV, Contamin L (2017). Quadruple-first line drug resistance in *Mycobacterium tuberculosis* in Vietnam: What can we learn from genes?. Genetics Evolution.

[4] Huyen MNT, Kremer K, Lan NTN (2013). Tuberculosis relapse in Vietnam is significantly associated with *Mycobacterium tuberculosis* Beijing genotype infections. The Journal of Infectious Diseases.

[5] World Health Organization (2020). Meeting report of the WHO expert consultation on the definition of extensively drug-resistant tuberculosis.

[6] Nguyễn ThỊ, Vân Anh (2012). DỊch tễ học phân tử Lao tại Việt Nam (2003-2009). Viện Vệ sinh dỊch tễ Trung ương, Hà NỘi.

[7] Huyen MNT, Buu TN, Tiemersma E (2013). Tuberculosis relapse in Vietnam is significantly associated with *Mycobacterium tuberculosis* Beijing genotype infections. The Journal of Infectious Diseases.

[8] World Health Organization (2023). Catalogue of mutations in Mycobacterium tuberculosis complex and their association with drug resistance.

[9] Nguyễn Huy Điện (2009). Nghiên cứu lâm sàng, cận lâm sàng tràn dỊch màng phổi do lao có xét nghiệm HIV(+) và kháng thuốc của *Mycobacterium tuberculosis*. Trường Đại học Y Hà NỘi.

[10] Yadav RN, Bhalla M, Kumar G (2022). Diagnostic utility of GenoType MTBDRsl assay for the detection of moxifloxacin-resistant *Mycobacterium tuberculosis*, as compared to phenotypic method and whole-genome sequencing. The International Journal of Mycobacteriology.

[11] Walker TM, Miotto P, Köser CU (2022). The 2021 WHO catalogue of *Mycobacterium tuberculosis* complex mutations associated with drug resistance: a genotypic analysis. The Lancet Microbe.

[12] Merker M, Blin C, Mona S (2015). Evolutionary history and global spread of the *Mycobacterium tuberculosis* Beijing lineage. Nature genetics.

